# Self‐Assembly of Bent‐Core Nematics in Nanopores

**DOI:** 10.1002/smll.202506651

**Published:** 2025-09-05

**Authors:** Andriy Z. Maksym, Anatoliy S. Andrushchak, Yaroslav Shchur, Bouchta Sahraoui, Przemysław Kula, Monika Lelonek, Mark Busch, Patrick Huber, Andriy V. Kityk

**Affiliations:** ^1^ Lviv National Polytechnic University 12 S. Bandery str. 79013 Lviv Ukraine; ^2^ Yukhnovskii Institute for Condensed Matter Physics of NASU 1 Svientsitskii str Lviv 79011 Ukraine; ^3^ University of Angers LPhIA, SFR MATRIX, 2 Bd. Lavoisier Cedex 01, 49045 Angers France; ^4^ Faculty of Advanced Technology and Chemistry Military University of Technology Warsaw 00‐908 Poland; ^5^ SmartMembranes GmbH Heinrich‐Damerow‐Str. 4 06120 Halle(Saale) Germany; ^6^ Institute for Materials and X‐ray Physics Hamburg University of Technology Denickestr. 15 21073 Hamburg Germany; ^7^ Centre for X‐ray and Nano Science CXNS Deutsches Elektronen‐Synchrotron DESY Notkestr. 85 22607 Hamburg Germany; ^8^ Faculty of Electrical Engineering Częstochowa University of Technology Al. Armii Krajowej 17 Częstochowa 42‐200 Poland

**Keywords:** liquid crystal nanocomposites, mesoporous alumina, mesoporous silica, nanoconfinement, optical polarimetry, twist‐bent nematics

## Abstract

Bent‐core nematic liquid crystals exhibit unique properties, including giant flexoelectricity and polar electro‐optic responses, making them ideal for energy conversion and electro‐optic applications. When confined in nanopores, they can stabilize chiral nanostructures, enhance polar order, and enable defect‐driven switching – offering potential in nanofluidics, sensing, and adaptive optics. The thermotropic ordering of the bent‐core dimer CB7CB confined in anodic aluminum oxide (AAO) and silica membranes with precisely engineered cylindrical nanochannels – ranging from just a few nanometers to several hundred nanometers–is examined. These well‐aligned nanochannels enable high‐resolution polarimetry studies of optical anisotropy, revealing how geometric confinement affects molecular organization and phase behavior. Under weak confinement, CB7CB forms a layered heterophase structure, with nematic, splay‐bent, and twist‐bent heliconical phases likely arranged concentrically. As confinement increases, a Landau‐de Gennes analysis shows that ordered phases are suppressed, leaving only a paranematic phase under strong spatial constraints. Remarkably, temperature‐dependent changes in optical birefringence under confinement closely resemble those seen under applied electric fields, revealing a parallel between geometric and electro‐optic effects. Overall, this work demonstrates how nanoconfinement allows one to systematically tailor the self‐assembly and optical behavior of bent‐core nematics, enabling novel functionalities in responsive and anisotropic materials.

## Graphical abstract

Bent‐core nematic liquid crystals like CB7CB exhibit tunable molecular organization and optical responses when confined in cylindrical nanochannels of anodic aluminum oxide and silica. The study investigates how confinement–from weak to strong–modulates phase behavior, revealing heliconical structures, paranematic states, and birefringence patterns. High‐resolution polarimetry and theoretical modeling uncover parallels between geometric constraints and electro‐optic effects, offering insights for responsive nanomaterials.

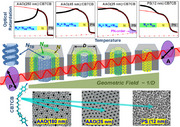

## 1 Introduction

Nanocomposites have emerged as a promising alternative to amorphous or crystalline materials in the design of novel advanced functional materials, often exhibiting properties not possessed by their conventional counterparts. The deposition of liquid crystals (LCs) in silica or anodic aluminum oxide (AAO) membranes yields very interesting nanocomposites, whose macroscopic optical and dielectric properties tend to be anisotropic and can be tailored by appropriate guest LC fillers, host matrix morphology and chemical surface grafting,^[^ ^1^, ^2^, ^3^, ^4^, ^5^, ^6^, ^7^, ^8^, ^9^, ^10^, ^11^, ^12^ ^]^ Optical properties, on the other hand, can be controlled by external fields, where interfacial interactions break symmetry rules, leading in certain cases to effects that are symmetry forbidden in their bulk components. The linear electro‐optic (Pockels) effect^[^ ^13^ ^]^ and the second‐order optical nonlinearity (SHG effect),^[^ ^14^ ^]^ recently demonstrated in nanoporous silica and AAO‐based inorganic–organic nanocomposites, can be considered as outstanding examples of such behavior, thus opening new perspectives for optoelectronic and photonic applications. From a fundamental point of view, the anchoring forces, defined by the specificity of the interfacial molecular interactions, together with the geometric constraint of the confined liquid crystal phase, exert a strong influence on the macroscopic physical properties of the LC nanocomposites as a whole.

Building on the foundational theoretical studies of Sheng, Poniewierski, and Sluckin,^[^ ^15^, ^16^ ^]^ along with the experimental investigations by Yokoyama^[^ ^17^ ^]^ on liquid crystals under semi‐infinite planar confinement, Kutnjak, Kralj, Lahajnar, and Žumer formulated a Landau–de Gennes free energy framework to describe the isotropic (I)‐nematic (N) transition in cylindrical confinement.^[^ ^18^, ^19^ ^]^ This model, commonly referred to as the KKLZ model, describes the molecular ordering of simple achiral rod‐like nematics under a geometric field σ associated with cylindrical confinement. According to their concept, confirmed in later polarimetric experiments,^[^ ^20^, ^21^, ^22^, ^23^ ^]^ the geometric field hypothetically scales inversely with the pore diameter (*σ*∝*D* ^−1^) and for symmetry reasons couples bilinearly with the orientational (nematic) order parameter. Thus, the behavior of the order parameter under geometrical constraints is similar to that observed for ferroelectrics and ferromagnets in external electric and magnetic fields, respectively, i.e., it is characterized by paranematic ordering and a critical point separating lines of discontinuous and continuous phase transitions between the paranematic and nematic states in the *σ* − *temperature* phase diagram.

However, the structure of liquid crystals in confined geometry can be more complex, which has been demonstrated in a number of experimental and theoretical studies. For example, discotic liquid crystals in cylindrical nanochannels show a competition between radial and axial columnar orders.^[^ ^24^, ^25^ ^]^ In hydrophilic nanochannels, a nematic shell of radially ordered columns affected by elastic splay deformations coexists with an orientationally disordered isotropic core. For these structures, the cylindrical boundary separating these phases is predicted to move from the channel walls to the channel center upon cooling, in accordance with the scaling behavior of the transition temperatures with the channel diameter. In contrast, the discotic LC confined in the hydrophobic nanochannels induces a quantized formation of annular layers consisting of concentric circularly bent columns,^[^ ^1^, ^26^, ^27^ ^]^ unknown in the bulk state.

The thermotropic behavior of nanoconfined cholesteric LCs also differs significantly from that of the bulk. Depending on the interfacial anchoring, it shows a radial or axial arrangement of the chiral LC molecules, which is indicated by a macroscopic optical birefringence and an optical activity of distinct positive and negative sign, respectively.^[^ ^28^ ^]^ For normal anchoring, a radial‐escape structure evolves upon cooling. For tangential anchoring, a large optical activity indicates a continuous paranematic to cholesteric transition with double twist helices aligned parallel to the long axes of the cylinders.

Due to their heterogeneity, confined LCs generally exhibit inhomogeneous dipolar dynamics with slow interface relaxation and fast core relaxation.^[^ ^29^, ^30^, ^31^, ^32^ ^]^ Surprisingly, under strong nanoconfinement, the dynamic electro‐optical response of ferroelectric LCs is accelerated by up to several orders of magnitude compared to the bulk,^[^ ^33^ ^]^ thereby greatly expanding the frequency range for their electro‐optical applications. Taken together, the structure of confined LCs and their associated static and dynamic properties generally result from the specificity of their molecular conformations, interfacial anchoring, and pore size/geometry.

Whereas the conventional rod‐like nematics are the simplest and technologically most applicable LC materials, liquid crystal research activities during the last decade have been refocused much on bent‐core LCs^[^ ^34^, ^35^ ^]^ as extraordinary materials both in fundamental and application aspects. Classical bent‐core liquid crystalline systems, also known as banana‐shaped mesogens, represent a distinct class of thermotropic liquid crystals characterized by their non‐linear, bent molecular geometry.^[^ ^36^ ^]^ Unlike conventional rod‐like (calamitic) mesogens, bent‐core molecules exhibit a unique combination of molecular asymmetry and dipolar interactions, leading to the formation of unconventional mesophases, such as the B1–B8 smectic phases and the elusive biaxial nematic phase. These mesophases are often associated with spontaneous polarization, chirality induction in achiral systems, and the emergence of polar domains, making bent‐core systems a rich platform for exploring ferro‐ and antiferroelectric liquid crystal behavior.^[^ ^37^, ^38^ ^]^ The discovery of these phases challenged traditional concepts in liquid crystal science and opened new pathways for developing materials with fast electro‐optic response, nonlinear optical properties, and switchable polar order.

A peculiar class of bent‐core molecules are cyanobiphenyl CB*n*CB (*n* = 7,9,11)^[^ ^39^, ^40^, ^41^ ^]^ or cyanoanilinebenzylidene CN‐*n*‐CN (*n* = 5,7,9)^[^ ^42^, ^43^ ^]^ dimers. They consist of two rigid cyanobiphenyl or cyanoanilinebenzylidene arms linked by an odd‐membered alkyl chain, making them conformationally flexible with a strong natural ability to pack into bent structures, although uniform bending in space is not allowed.

**Figure 1:**
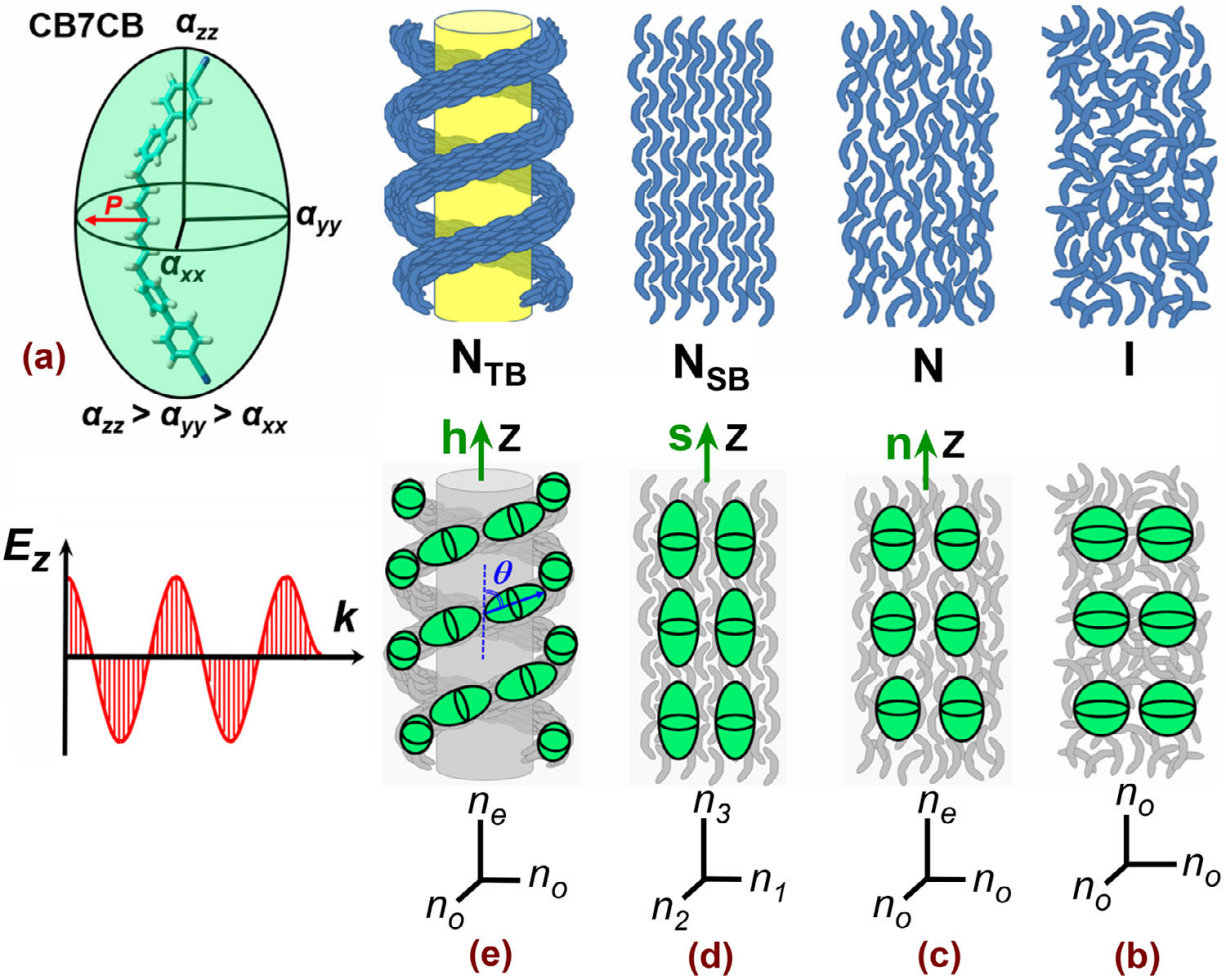
Liquid‐crystalline phases and birefringence characteristics of bent‐core nematics. a) Equilibrium bent‐core molecular conformation of CB7CB and corresponding molecular polarizibility ellipsoid describing its anisotropy. b–e) Sketches of the molecular self‐assembly in different LC states formed by arrangement of bent‐core molecules: b) ‐ totally isotropic (I) phase; c) ‐ uniaxial nematic (N) phase; d) ‐ locally and macroscopically biaxial splay‐bend nematic (N_*SB*_) phase; e) ‐ locally biaxial and macroscopically uniaxial twist‐bend nematic (N_*TB*_) phase. Green spheres and ellipsoids indicate the local optical polarizability anisotropy.

Analyzing this inconsistency, Meyer^[^ ^34^ ^]^ and subsequently Dozov^[^ ^35^ ^]^ and Memmer^[^ ^44^ ^]^ theoretically predicted the possibility of realizing two spatially inhomogeneous LC states characterized by combinations of either twist‐bend or splay‐bend spontaneous distortions corresponding to the nematic twist‐bend (N_*TB*_) and nematic splay‐bend (N_*SB*_) phases, respectively. For both LC states, the twist or splay is required to provide spatially uniform bending, resulting in spatially modulated LC molecular configurations. The N_*TB*_ structure is described by a conical twist‐bend helix, where the helical axis is the mean optical axis of the medium, see **Figure** **1**. Surprisingly, the N_*TB*_ phase, consisting of achiral bent molecules, is chiral with doubly degenerate chirality, i.e., in the bulk state it consists of right and left monochiral domains.^[^ ^35^ ^]^ The N_*SB*_ phase, on the other hand, is achiral and can be described in the simplest approximation by a linearly polarized plane wave with director distortion.

Taken together, the self‐assembly of achiral bent‐core molecules can lead to one of three nematic states with different macroscopic symmetry: i) conventional uniaxial and achiral N‐phase, ii) locally biaxial but macroscopically uniaxial doubly degenerate chiral N_*TB*_ phase, and iii) both locally and macroscopically biaxial and achiral N_*SB*_ phase, see Figure 1. The transition from the conventional uniform to the periodic twist‐bend or splay‐bend nematic state is associated with a change in the sign of the bending elastic constant *K*_3_ from positive to negative. The stability of the relevant phases, on the other hand, is defined by the ratio of the splay *K*_1_ to the twist *K*_2_ elastic constants.^[^ ^35^, ^45^ ^]^ For *K*_2_ < *K*_1_/2 the heliconical twist‐bend configuration is realized, otherwise the splay‐bend oscillation becomes energetically more favorable. In practice, however, the N_*TB*_ phase is usually realized for bent dimers, indicating a low *K*_1_/*K*_2_ ratio that ensures their thermodynamic stability. However, an N_*TB*_ to N_*SB*_ transition can be induced by an external electric field applied parallel^[^ ^46^ ^]^ or perpendicular^[^ ^47^, ^48^ ^]^ to the helix axis, which in practice results in a giant electro‐optical response.^[^ ^49^ ^]^ The N_*SB*_ phase can also be stabilized under topological confinement, such as a defect wall between two N_*TB*_ domains of opposite chirality,^[^ ^46^ ^]^ or surface‐induced under extremely strong anchoring near the interface.^[^ ^50^, ^51^ ^]^ For this reason, the phase behavior of bent‐core LCs under nanoscale confinement is of great interest, as geometric confinement combined with specific interfacial anchoring is expected to play an increasingly important role in stabilizing relevant confined states or heterophase formations resulting from their molecular self‐assembly under such combined conditions.

For bent‐core LCs, the number of available experimental and theoretical studies under confinement is rather limited compared to other LC materials. The reported X‐ray^[^ ^52^ ^]^ and dielectric^[^ ^53^ ^]^ experiments were performed using AAO as the host medium with specific channel diameters in the submicron range, 100 and 200 nm, respectively, i.e., limited to a regime of weak spatial confinement. Here, we combine alumina and silica mesoporous membranes to cover an exceptionally wide range of channel diameters from a few to several hundred nanometers. This provides the opportunity to study the phase behavior of the bent‐core nematic under weak, moderate, and strong geometric confinement. The specific morphology of mesoporous alumina and silica networks, consisting of parallel aligned cylindrical nanochannels, allows the study of optical anisotropy related to the arrangement of bent‐core molecules inside the nanochannels using high‐resolution optical polarimetry.

## 2 Experimental Section

The dimer 1″, 7″‐bis(4‐cyanobiphenyl‐4′‐yl)heptane (CB7CB) was used as a guest LC component for the preparation of silica‐ and alumina‐based LC nanocomposites. Compared to other members of the homologous CB*n*CB series, CB7CB was the best characterized bent‐core dimeric mesogen in the literature, which justifies this choice. The chemical structure of the CB7CB molecule (Figure 1) consisted of two identical cyanobiphenyl moieties linked by a seven‐membered alkyl chain. The odd‐membered flexible spacer defines its bent shape as the most stable molecular conformation in the ground state. The molecular polarizability tensor, evaluated by the semiempirical quantum chemical AM1 method,^[^ ^54^ ^]^ was characterized by the largest tensor component parallel to the alkyl chain of the CB7CB molecule (*α*_*zz*_ > *α*_*yy*_ > *α*_*xx*_, see sketch in Figure 1), which caused a positive optical anisotropy in the bulk nematic state (δ*n* = *n*_*o*_ − *n*_*e*_ > 0). A large dielectric anisotropy (Δ*ϵ* > 0),^[^ ^55^ ^]^ on the other hand, gives rise to a strong response to the applied field, which was important for possible electro‐optical applications. The CB7CB was synthesized at the Warsaw Military University of Technology following the method described in Ref. [56]. In the first step the inner part of the bimesogenic structure was synthesized via Friedel‐Crafts acylation followed by hydrazine reduction of the diketone. In the next step of forming the final compounds CB7CB, the dibromoderivative was cross‐coupled with 4‐cyanophenylboronic acid or its cyclic ester, obtained via sequential halogen‐magnesium‐boron exchange. The Suzuki‐Miyaura cross‐coupling was performed using palladium acetate and bulky phosphine ligand CataXium in conventional water acetone solvent system using potassium carbonate as base. Upon slow cooling (0.2 Kmin^−1^) from the isotropic phase, the bulk CB7CB undergoes two successive phase transitions before solidification: to the N phase at *T*_*IN*_ = 390 ± 0.5K and further to the N_*TB*_ phase at *T*_*NTB*_ = 377 ± 0.5K, in reasonable agreement with previous studies.^[^ ^39^, ^53^, ^57^, ^58^ ^]^ This independently confirms the purity of the synthesized bent‐core dimer.

The mesoporous silica membranes *p*SiO_2_ (hereafter *PS*), had been prepared by electrochemical anodic etching of heavily boron doped silicon wafers, followed by their thermal oxidation for 12 h at *T* = 800°C in standard atmosphere. By varying the etching time, a series of *PS* membranes with average channel diameters *D* = 6.0 ± 0.5 nm (porosity *P* = 13 ± 1%, thickness *h* = 120 ± 5µm), *D* = 8.0 ± 0.6nm (*P* = 20 ± 2%, *h* = 160 ± 8µm), and *D* = 12.0 ± 1.0 nm (*P* = 40 ± 3%, *h* = 270 ± 10 µm) was obtained, as verified by recording volumetric N_2_‐sorption isotherms at *T* = 77 K.

The nanoporous alumina membranes *p*Al_2_O_3_ (hereafter *AAO*) were purchased from Smart Membranes GmbH (Halle, Germany). The specifications of the *AAO* membranes were verified by analysis of SEM (scanning electron microscope) images, which yielded the following average channel diameters and corresponding porosities: *D* = 25.0 ± 2.0 nm (*P* = 14 ± 1%, *h* = 105 ± 5µm), *D* = 35 ± 3 nm (*P* = 17 ± 1.5%, *h* = 100 ± 5µm), *D* = 45.0 ± 4.0 nm (*P* = 26 ± 2%, *h* = 100 ± 5µm), *D* = 60 ± 5 nm (*P* = 28 ± 2%, *h* = 140 ± 5µm), *D* = 180.0 ± 10.0 nm (*P* = 13 ± 1.5%, *h* = 105 ± 5µm), *D* = 250 ± 15 nm (*P* = 24 ± 2%, *h* = 55 ± 5µm).

**Figure 2:**
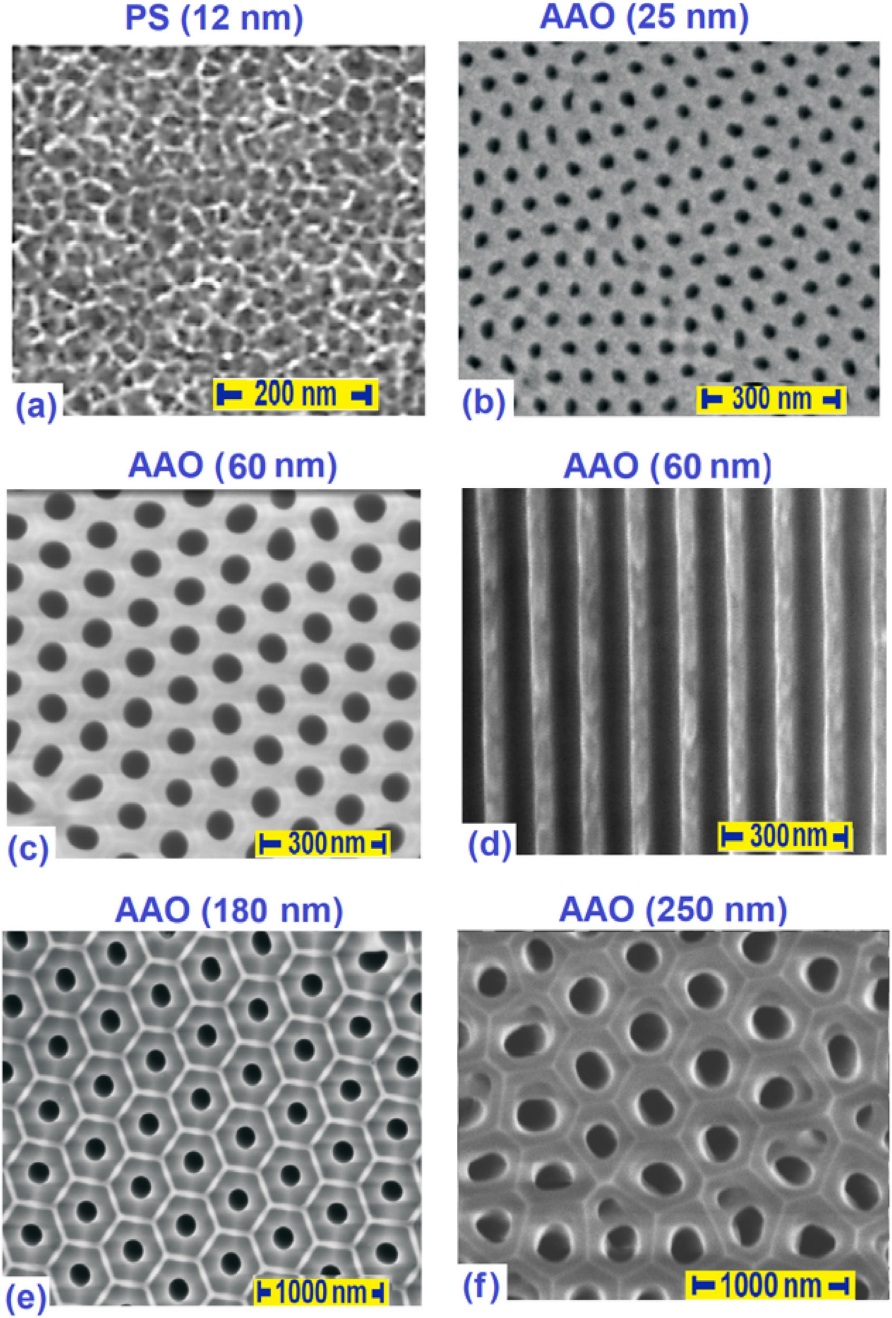
Structure of mesoporous host materials. a) Scanning electron micrographs of the mesoporous silica (*PS*) and b–f) mesoporous anodic aluminum oxide (*AAO* membranes used for preparation LC nanocomposites *PS*:CB7CB and *AAO*:CB7CB, respectively. Panel (a),(b),(c),(e) and (f) contain top views, whereas panel (d) shows a side view of an *AAO* membrane cleaved parallel to the nanochannel axes.

The mesoporous *PS* and *AAO* membranes were first annealed at 440 K for about 15 min to remove the adsorbed water from the channel walls. They were then completely filled by capillary action (spontaneous imbibition) of CB7CB LC in the isotropic phase at *T* ≈400 K.^[^ ^59^ ^]^ The composites prepared in this way, hereafter referred to as *PS*:CB7CB and *AAO*:CB7CB, combine an inorganic silica or alumina framework, which provided their mechanical stiffness, and a nematic LC, which introduces an optical anisotropy related to the specific self‐assembly of the guest bent‐core molecules, which was the subject of the experimental studies. Nevertheless, the specificity of the host matrix morphology seems to be a crucial aspect of their optical anisotropy and therefore deserves a more detailed characterization. The SEM image shown in **Figure** **2**a is typical for *PS* membranes and clearly shows the lateral irregularity of the mesoporous channel network. In contrast, the *AAO* templates obtained by two‐step electrochemical anodization were characterized by a hexagonally ordered morphology, see Figure 2,b,c,e,f. However, despite the difference in lateral morphology, both *PS* and *AAO* form an assembly of parallel cylindrical nanochannels oriented perpendicular to the membrane surface, as shown in Figure 2d. This results in their uniaxial macroscopic optical anisotropy with the optical axis parallel to the long channel axis, which fundamentally distinguishes them from optically isotropic random porous media such as Vycor glass. Filling the channels with isotropic liquids changes the optical anisotropy associated with geometric birefringence. In the case of anisotropic guest liquids, an additional influence on the macroscopic optical birefringence results from the orientational and/or positional molecular ordering in the nanochannels, in particular from the molecular self‐assembly.

**Figure 3:**
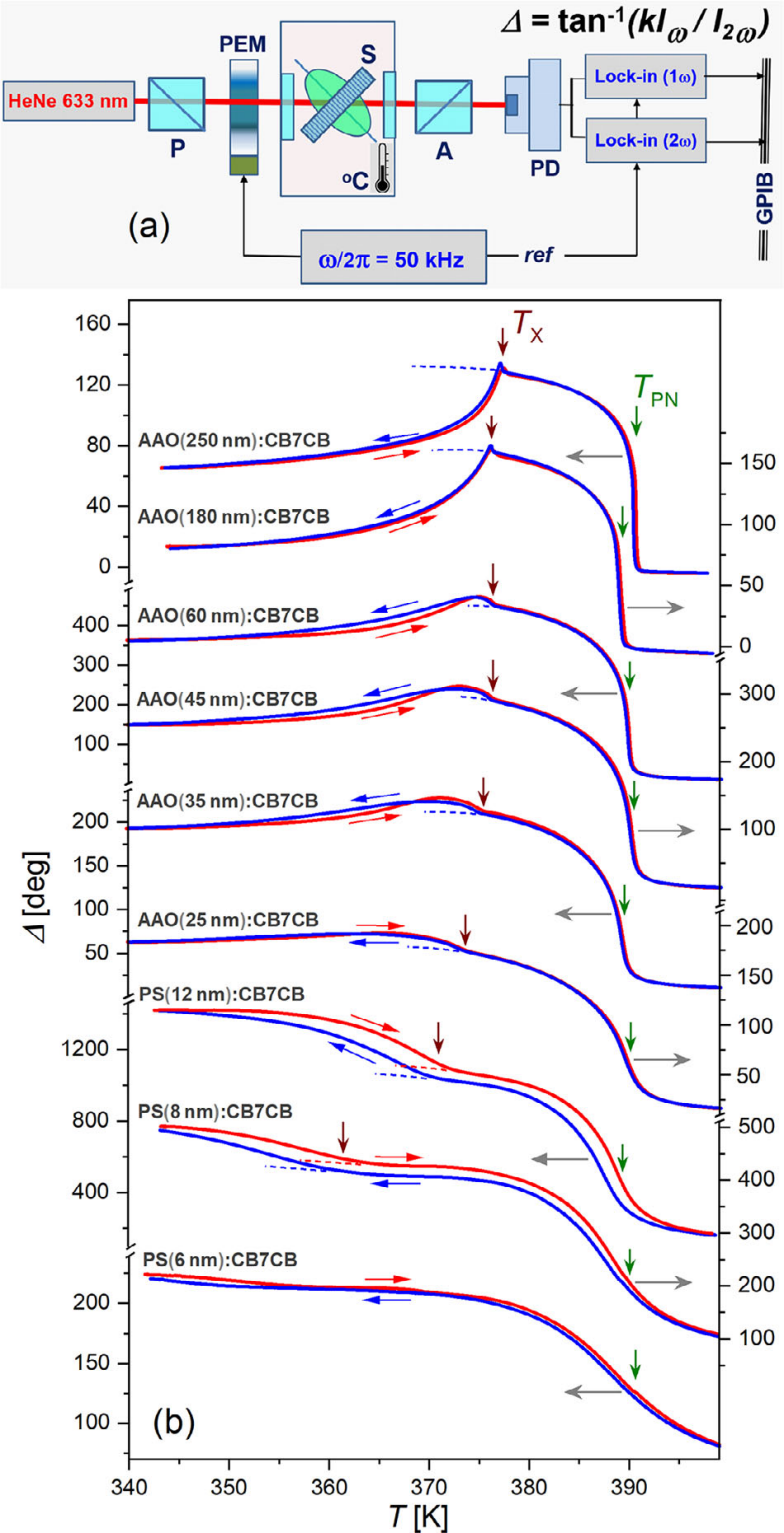
Birefringence experiments on thermotropic liquid crystal ordering. a) Sketch of the optical polarimetry setup. b) Temperature‐dependent optical retardation Δ measured during heating (red) and cooling (blue) of a series of *AAO*:CB7CB and *PS*:CB7CB nanocomposites.

Accordingly, high‐resolution optical polarimetry had been used to study the temperature variation of the optical birefringence of *PS*:CB7CB and *AAO*:CB7CB nanocomposites. The self‐made optical polarimetry setup uses a photoelastic modulator (PEM) PEM‐90 (Hind Instruments), see **Figure** **3**a, and is similar to that used in several previous experiments.^[^ ^20^, ^28^ ^]^ The PEM and the sample (S) were placed between the crossed polarizer (P) and the analyzer (A). The modulated laser light (λ = 633 nm), caused by time modulated phase retardation (δ(*t*) = *A*_0_sin (ω*t*), *A*_0_ = 0.383λ, ω/2π = 50 kHz), was detected by the photodiode (PD) and then analyzed by pairs of lock‐in amplifiers that simultaneously extract the amplitudes of its first (*I*_1ω_) and second (*I*_2ω_) harmonics. In such a polarimetric scheme, the static phase retardation Δ induced by the sample is given by

(1) $$\Delta =\tan^{-1}\left(kI_{1\omega }/I_{2\omega }\right)$$

where the effective factor *k*, defined by the frequency response characteristics of the photodetector and the ratio of the Bessel function values *J*_2_(*A*_0_)/*J*_1_(*A*_0_), was determined directly within the calibration procedure using a standard quarter‐wave retarder (a λ/4 plate). This polarimetry technique provides an accuracy of optical retardation measurements better than 0.005°. It should be noted that for a fixed angle of incidence *α* the optical birefringence Δ*n* scales linearly with the retardation induced by the sample, i.e., Δ*n*∝Δ. The retardation Δ, on the other hand, increases with *α*. Therefore, in order to access the uniaxial optical anisotropy, the sample was tilted by an angle *α* = 36° with respect to the optical axis, which ensures an adequate measurement sensitivity to the birefringence changes with a moderate reduction of the transmitted light caused by its reflection at the membrane surfaces. The sample was placed in a temperature‐controlled optical cell operated by a Lakeshore‐340 controller with a temperature control accuracy of 0.01 K. Polarimetric measurements were performed by slowly ramping the sample temperature with a heating/cooling rate of 0.15 K·min^−1^.

For rod‐like molecules, both the optical birefringence Δ*n* and the associated optical retardation Δ were proportional to the orientational order parameter *Q* = $\frac{1}{2}\left\langle 3\cos^{2}\phi -1\right\rangle$, where *ϕ* was the angle between the long axis of a single molecule and a direction of preferred orientation of that axis, the director **n**. The brackets denote an averaging over all molecules considered. For relevant representatives, such as the cyanobiphenyl monomers *n*CB (*n* = 5‐10), the maximum molecular polarizability nearly coincides with the long axis of these molecules, hence their optical anisotropy in the bulk N‐state was positive, i.e., *n*_*e*_ > *n*_*o*_. In the case of the bent‐core CB7CB molecule in its equilibrium ground state, the long axis of molecular polarizability was parallel to the seven‐membered alkyl chain, although the cyanobiphenyl monomers, which contribute most to the polarizability, were attached to the alkyl chain at a considerable angle (≈33°), as shown in Figure 1a based on the results of semiempirical quantum chemical calculations.^[^ ^54^ ^]^ Dimer LC CB7CB, on the other hand, similarly to its monomer counterpart, exhibits positive dielectric and optical anisotropy in the bulk N‐phase, which had been demonstrated in recent experimental studies.^[^ ^55^ ^]^ Accordingly, it could be concluded that the axis parallel to the alkyl chain of the CB7CB molecule represents the molecular axis in the sense of the fundamental order parameter definition. This means that the conventional N state was associated with a self‐organized arrangement of bent‐core molecules, whose longest polarizability axes approach a preferred orientation along the director direction, where the local polarizability was spatially uniform, as schematically shown in Figure 1c.

The N_*TB*_ phase, on the other hand, represents a spatially inhomogeneous state described by a heliconical director field. In the nanochannels of the host matrix, it likely forms N_*TB*_ clusters of opposite chirality that were in statistical equilibrium. Consequently, the corresponding nanocomposites exhibit no macroscopic optical activity, a finding also confirmed in the experiments. The clusters of opposite chirality, on the other hand, equally contribute to the macroscopic optical birefringence. Due to the heliconical precession of the local polarizability, see sketched by green ellipsoids in Figure 1d, its longest axis appears to be tilted out with respect to the helical axis **h**. As a consequence, the macroscopic optical anisotropy was uniaxial, whereas the optical birefringence that characterizes it should decrease at the N→N_*TB*_ phase transition, which had indeed been observed in optical polarimetry experiments.^[^ ^46^, ^60^ ^]^ In the N_*SB*_ phase, the director oscillates in one plane, i.e., it is a linearly polarized wave of alternating splay and bend distortions of the nematic director field, Figure 1c. In contrast to the N or N_*TB*_ phase, the N_*SB*_ was both locally and macroscopically biaxial, i.e., its bulk optical anisotropy was characterized by three refractive indices. However, when considering the *PS*:CB7CB and *AAO*:CB7CB nanocomposite membranes, it should be noted that the tubular morphology of their host matrix had a strong influence on the optical anisotropy studied by the optical polarimetry technique. It was obviously strongly dependent on the molecular arrangement with respect to the channel geometry, but the local lateral anisotropy in adjacent channels was uncorrelated. The macroscopic (averaged) anisotropy of nanocomposite membranes was therefore always uniaxial with the optical axis parallel to the long channel axis, i.e., perpendicular to the membrane plane. Nevertheless, based mainly on the results of polarimetric measurements, their combined qualitative‐quantitative analysis allows to gain deep insights into the understanding of the self‐assembly of mesogenic bent‐core molecules caused by successive phase transitions under nanoconfinement.

There were no signs of degradation of the nanocomposites. Furthermore, the LC wets the inner surfaces of the AAO membranes preferentially. Therefore, presumably, any leakage was observed.

## 3 Results and Discussion

### 3.1 Evolution of Collective Orientational Order as a Function of Temperature and Confinement

By combining alumina and silica host membranes with channel diameters ranging from 6 to 12 nm (*PS*) and from 20 to 250 nm (*AAO*), our study covers different geometric confinement regimes, which turn out to have a strong influence on the phase behavior of the bent‐core nematic confined in nanochannels. In Figure 3b, we show the optical retardation Δ versus temperature *T* for the series of LC nanocomposite membranes *PS*:CB7CB and *AAO*:CB7CB measured at constant incident angle (*α* = 36°) during heating and subsequent cooling.

For large diameter channels, e.g. *D* = 250 or 180 nm, corresponding to a weak confinement regime, the temperature dependence of Δ closely resembles the behavior of the bulk CB7CB,^[^ ^46^, ^60^ ^]^ revealing two characteristic temperature points associated with successive structural transformations, marked by arrows in Figure 3b and designated as *T*_*PN*_ and *T*_*x*_. The untreated surface of both alumina and silica membranes enforces planar anchoring, while the elongated geometry of the cylindrical pores additionally results in a preferred orientation of the molecules along the pore axes. Therefore, the high temperature state (*T* ⩽ *T*_*PN*_) is paranematic (PN) with a heterogeneous phase structure of the confined LC consisting of an isotropic core and a nematically ordered near‐interface layer. The transition from the PN to the N state (*T* = *T*_*PN*_) is accompanied by a jump‐like increase in the optical retardation Δ, while the positive sign of the excess retardation in the nanoconfined N phase clearly indicates the axial molecular ordering. The transition at lower temperature, i.e. at *T* = *T*_*x*_, is characterized by a small but distinct sharp increase in the retardation Δ, followed by a significant continuous decrease observed upon further cooling.

Based on the similarity of such behavior in the region of this transition to that of bulk CB7CB reported in Ref. [46, 60], we suggest that the confined LC state below *T*_*x*_ is the N_*TB*_ state. Assuming that the helical axis is parallel to the channel axis, the decrease in optical retardation indeed confirms the tilting of the molecules upon formation of the heliconical structure. In fact, the self‐assembly of the bent‐core molecules under nanoconfinement is more complex, generally forming a multiphase system, although for large channel diameters the N_*TB*_ fraction dominates, which is the reason for the observed temperature evolution of the optical retardation in this temperature range. We will return to this issue in the discussion below.

For moderate confinement, i.e., channel diameters 25 ⩽ *D* ⩽ 60 nm, the variation of the optical retardation becomes more and more gradual with decreasing pore diameters in the vicinity of both phase transition temperatures. The smearing of the jump‐like behavior in the region of the PN‐N transition is accompanied by an enhancement of the paranematic ordering with a characteristic tail extending well beyond *T*_*PN*_, which seems to be typical for many rod‐like nematics, including also the mesogen 7CB, i.e., the monomer counterpart of the CB7CB dimer. The decrease in optical anisotropy below *T*_*x*_, apparently associated with the formation of a heliconical structure and clearly observed in the bulk state or under weak confinement, appears to be much less pronounced under moderate confinement. The temperature behavior of the optical retardation exhibited by *AAO*(45 nm):CB7CB or *AAO*(35 nm):CB7CB LC nanocomposites, see Figure 3b, is very similar to the behavior of bulk CB7CB under a strong electric field applied along the helical axis.^[^ ^46^ ^]^ Indeed, this comparison reveals a very interesting analogy between the electric and geometric fields, which act in a similar manner and tend to unwind the helical structure. The authors of Ref. [46] suggest that the electric‐field‐induced phase is the N_*SB*_ state, based on the similarity of the birefringence behavior to that exhibited by the topological N_*SB*_ wall stacked between the two N_*TB*_ domains of opposite chirality. However, a certain decrease of the optical retardation followed by its increase below *T*_*x*_ may indicate that the N_*TB*_‐fraction is rather not completely suppressed under both confinement and electric field.

In the case of strong confinement, i.e., channel diameters 8 ⩽ *D* ⩽ 12 nm, we still observe two successive phase transitions. However, the character of the retardation behavior near and below *T*_*x*_ is different from that observed for moderate confinement. Below *T*_*x*_ the Δ(*T*) dependences show only an increase of the saturation, i.e., no decrease of its value. This finding indicates that the tilting of the bent‐core molecules with respect to the long channel axis, which would indicate the formation of a helical structure, is negligible. One can therefore conclude that strong confinement completely suppresses the N_*TB*_ phase and eventually stabilizes the N_*SB*_ phase fraction in the core region of the channel filling.

Extremely strong confinement (*D* = 6 nm) results in complete suppression of the low temperature phase. The self‐assembly of the bent‐core CB7CB molecules is strongly favored by the paranematic interfacial ordering, which significantly affects the transition to the nematic state. As a result, only a very gradual phase transition from the PN to the N state is observed, extending over a temperature range of tens of degrees.

### 3.2 Landau‐De Gennes Free Energy Analysis of the Paranematic‐To‐Nematic Phase Transition

The spatial constraint has no significant effect on the PN‐to‐N phase transition temperature *T*_*PN*_ even under extremely strong confinement. In contrast, the transition temperature *T*_*x*_, apparently related to the N‐to‐N_*SB*_ structural transformation in the core region of the pore filling, shows a downward shift with decreasing channel diameter, which accelerates significantly at *D* < 25 nm. There are fundamental reasons for such a difference. In the case of the N‐state with axial molecular arrangement, the cylindrical geometric confinement and the associated bending distortion do not break its axial symmetry. According to the KKLZ model,^[^ ^18^, ^19^ ^]^ the bilinear coupling between the nematic order parameter *q* and the geometric field *σ*∝*D* ^−1^ (see Equation (2)), leads to an increase of the *T*_*PN*_ temperature under confinement, while the quenched disorder effect, described by the *κ* *q* ^2^ term, does the opposite. In practice, these contributions nearly compensate each other, so that the confinement shift of the PN‐to‐N transition is usually insignificant, as has been demonstrated for a number of rodlike mesogens.^[^ ^22^, ^23^ ^]^

In the case of the N_*SB*_ phase, on the other hand, the biaxial symmetry is broken under the bend/splay distortion caused by the cylindrical geometric constraint. Among the possible configurations, one can consider molecular self‐assemblies in which the long molecular axes, associated with the highest optical polarizability, are nearly parallel to the channel axis, while their shortest molecular axes can be oriented to form radial, log‐pile, or circular concentric configurations. In all cases, the bending and/or splay distortion in the lateral membrane plane due to the cylindrical confinement will increase the free energy of the confined N_*SB*_ state. This will inevitably stabilize the N phase, shifting the N to N_*SB*_ transition significantly downward with temperature. Due to the biquadratic coupling between the order parameter and the splay and/or bend distortion, the temperature shift of this transition is expected to be ∝*D* ^−2^ (see e.g.,^[^ ^61^ ^]^ and references therein), i.e. *T*_*x*_ shows an accelerating decrease as one approaches the regime of strong confinement.

Under weak and moderate confinement, both phase transitions are accompanied by only a small temperature hysteresis (≈0.1–0.3 K), which increases to ≈4–5 K under strong confinement. Nevertheless, the optical retardation measured during subsequent heating and cooling cycles shows almost identical temperature evolution. For this reason, its further quantitative analysis is limited only by the Δ(*T*) dependence recorded during cooling.

**Figure 4:**
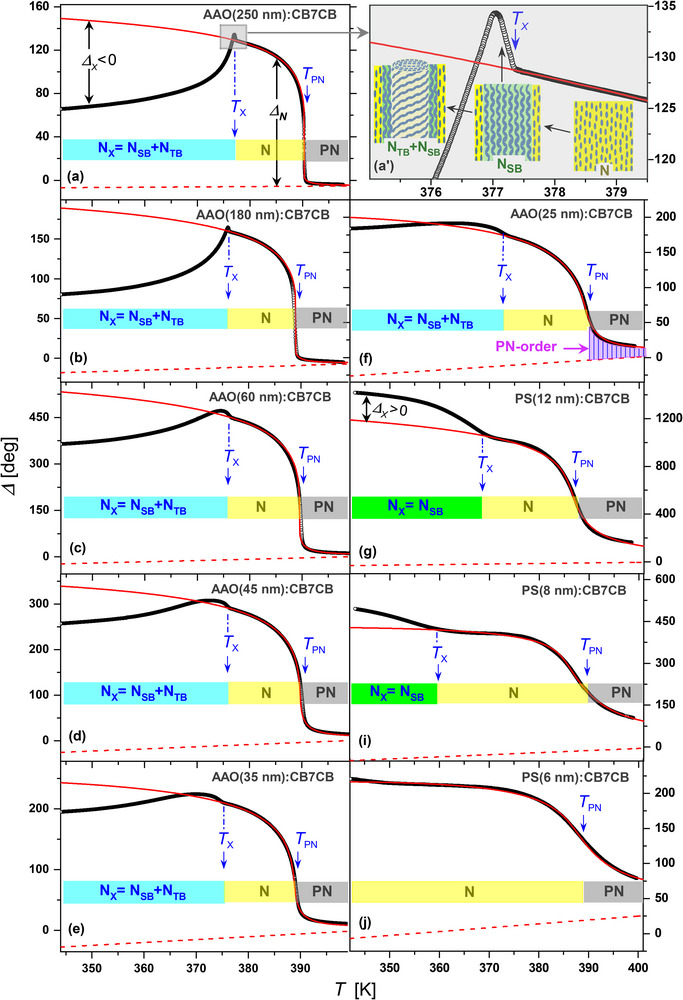
Comparison between optical experiment and free energy modeling. The temperature dependence of the optical retardation Δ measured on cooling (gray symbols) and their KKLZ fits (solid lines, red color) in the nematic phase extrapolated to the N_*SB*_/N_*TB*_ multiphase region (T<*T*_*x*_) for a series of *AAO*:CB7CB and *PS*:CB7CB nanocomposites, see labels. Dashed lines (red color) represent thermooptic baselines extracted within the same fitting procedure.

**Figure** **4** shows the temperature dependence of the measured optical retardation recorded during cooling (symbols) along with the KKLZ fits of the retardation data in the confined PN and N states (*T* ⩾ *T*_*x*_) and extrapolated to the low temperature LC state temperature region, i.e., *T* < *T*_*x*_ (solid red curves). The orientational ordering within the cylindrical channels, described by the nematic order parameter *q*, leads to an excess retardation, Δ_*N*_∝*q* (see Figure 4a), which scales with the porosity of the nanoporous host membrane and appears on the background of the onset retardation caused by the geometric birefringence, Δ*n*_*g*_, resulting from the tubular morphology of the mesoporous substrate.^[^ ^20^, ^21^, ^22^, ^23^ ^]^ Accordingly, a simple linear relationship between the measured optical retardation and the nematic order parameter *q*_*o*_ is maintained for the nanocomposite, provided that the geometric retardation onset is subtracted. Due to the small difference between the refractive indices of the mesoporous alumina or silica host matrix and the guest LC CB7CB, the geometric birefringence as well as the associated onset retardation are relatively small and weakly dependent on temperature, mainly defined by the changing refractive indices of the host/guest materials and the porosity due to thermal contraction of the porous matrix during cooling. To a good approximation, it can be defined by a linear temperature dependence and then determined within the fitting procedure,^[^ ^22^, ^23^ ^]^ see dashed red lines in Figure 4.

While the orientational molecular ordering at the bulk isotropic to nematic phase transition can be well described by a Landau‐de Gennes theory, the KKLZ model extends its application to spatially confined nematic states. The orientational ordering within the KKLZ approach is defined by the scaled order parameter *q* = *Q*/*Q*(*T*_*IN*_), i.e., the order parameter normalized by the value of its jump at the bulk isotropic‐to‐nematic phase transition temperature *T*_*IN*_. The dimensionless free energy density of a nematic LC in the confined geometry defined by the geometric field *σ*∝*D* ^−1^ is given by

(2) $$f=tq^{2}-2q^{3}+q^{4}-q\sigma +\kappa q^{2}+h\left(q^{n}\right)$$

where *t* = (*T* − T*)/(*T*_*IN*_ − T*) is the dimensionless reduced temperature, T* is the effective temperature, the *κ* term comes from the contribution of quenched disorder effects. *h*(*q* ^*n*^) represents the contribution of higher‐order power terms in *q*, which provides a more accurate description of the nematic order parameter in a wide temperature range. Minimizing Equation (2) with respect to *q* gives its equilibrium value *q*_*o*_.

The free energy expansion of the classical KKLZ model (*h*(*q* ^*n*^) = 0) is limited up to fourth‐order terms in *q*. Within this approach the PN‐to‐N transition occurs at *t*_*PN*_ = 1 + σ − κ and is discontinuous as long as the geometric field *σ* is smaller than its critical value *σ*_*c*_ = 0.5. Above this value, the PN‐to‐N transition becomes continuous. Interestingly, within the modified KKLZ approach (*h*(*q* ^*n*^) ≠ 0), described particularly by Equation (2), the value *σ*_*c*_ appears to be indeed smaller, as will be discussed below. *σ* and *κ* terms give opposite contributions to the temperature shift of this phase transition under confinement, so the practical independence of *T*_*PN*_ on the channel diameter *D* observed in the experiment indicates that their respective contributions should compensate each other. The orientational order inside the pores leads to an excess birefringence, Δ_*N*_∝*q*_*o*_. The difference *T*_*IN*_ − T* = 2.2 K, which defines the proximity of the nematic system to the tricritical point, was determined by analyzing the excess birefringence behavior of CB7CB in weak confinement (*D* = 250 nm) and was fixed to this specific value in all subsequent fitting procedures.

**Figure 5:**
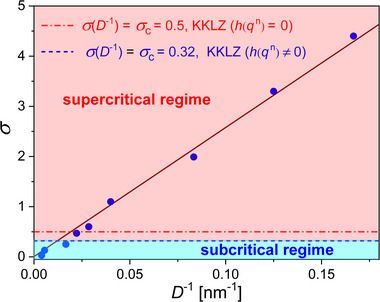
Strength of the surface ordering field σ versus pore diameter *D* ^−1^ extracted within the KKLZ‐analysis of the Δ(*T*)‐dependences measured on a series of AAO:CB7CB and PS:CB7CB nanocomposites. The horizontal broken line (blue), resulting from the analysis based on the modified KKLZ approach (*h*(*q* ^*n*^) ≠ 0), separates the subcritical and supercritical regions. The horizontal dashed line (red online) indicates the critical value *σ*(*D*) = *σ*_*c*_ = 0.5 predicted by the classical KKLZ approach (*h*(*q* ^*n*^) = 0).

The solid red line in Figure 4 represents the best fit of the measured retardation in the confined nematic phase obtained within the KKLZ model based on a free energy expansion (2). The inclusion of higher order terms (up to 10th order) well describes the saturation of the order parameter at lower temperatures. The thermo‐optical contribution (dashed lines, red online color), approximated by a linear dependence, is obtained within the same fitting procedure. The geometric order field extracted from the fits shows a linear dependence on the inverse channel diameter, i.e., *σ*∝*D* ^−1^, see **Figure** **5**. The critical geometric field, which corresponds to the critical channel diameter *D*_*c*_ = 79.0 nm [*σ*_*c*_ = *σ*(*D*_*c*_) = 0.32], separates the region of discontinuous phase transitions (*σ* < *σ*_*c*_, *D* > *D*_*c*_, subcritical regime) from the region of continuous phase transitions (*σ* ⩾ *σ*_*c*_, *D* ⩽ *D*_*c*_, supercritical regime). One can see that extracted from the fitting analysis *σ*_*c*_ value within the modified KKLZ approach (*h*(*q* ^*n*^) ≠ 0) is lower of the value *σ*_*c*_ = 0.5 predicted by the classic KKLZ model (*h*(*q* ^*n*^) = 0).

The observed optical retardation behavior of the bent core mesogen CB7CB in the region of the confinement‐constrained PN‐to‐N transition does not differ much from that previously reported for several simple rod‐shaped mesogens,^[^ ^20^, ^21^, ^22^, ^23^ ^]^ despite a considerable difference in their molecular structure. However, an adequate description of the orientational order in the nematic phase seems to be important from the point of view of the interpretation of the low‐temperature phase transition, since confined N_*TB*_ or N_*SB*_ or their combined hybrid states appear due to spontaneous molecular reorganization of the parent nematic phase; therefore, the extrapolated behavior of the nematic order parameter and the associated excess retardation Δ_*N*_(*T*) (*T* ⩽ *T*_*x*_) will be used as a reference in further analysis.

### 3.3 Splay‐Bend to Twist‐Bend Phase Transitions and Phase Coexistence

In the case of weak confinement (*D* = 250 nm, Figure 4a), the overall temperature behavior of the optical retardation is similar to that of the bulk state reported in several recent works.^[^ ^46^, ^60^ ^]^ On cooling, the excess retardation Δ_*x*_ with respect to the retardation extrapolated from the nematic phase becomes significantly negative well below *T*_*x*_. Apparently, this behavior is consistent with the formation of the heliconical structure of the N_*TB*_ phase with the helix axis parallel to the long channel axis. In the case of the *AAO*:CB7CB nanocomposite sample, a significant reduction in the measured optical retardation is apparently caused by tilting of the bent‐core molecules with respect to the long channel axis, with both right‐handed and left‐handed chiral regions (clusters) of the confined CB7CB mesogen contributing statistically equally to the macroscopic optical anisotropy studied by polarimetry. However, such an interpretation seems to be inconsistent in the vicinity of the characteristic phase transition temperature *T*_*x*_, as shown in detail in the extended view of Figure 4a'. The optical retardation unexpectedly shows here a small but distinct abrupt increase, obviously different from the birefringence behavior of bulk CB7CB reported by Tuchband *et al.* ^[^ ^60^ ^]^.

What could be the reason for such behavior? While an abrupt increase of the already saturated orientational order is rather unlikely, a positional reorganization of the bent‐core molecules leading to a further increase of the optical anisotropy is possible. In particular, such behavior is characteristic for bulk and confined nematic‐to‐smectic transitions^[^ ^21^ ^]^ or cholesteric‐to‐smectic transitions,^[^ ^28^ ^]^ where the increase of optical birefringence is caused by smectization of nematic order. The formation of smectic layers causes a stronger packing of the molecules, which is one of the main reasons for the increasing optical anisotropy. However, X‐ray scattering experiments did not confirm the formation of smectic layers in this temperature region. For this purpose, we carried out experiments on the AAO membrane with the largest pore diameter, i.e., 250 nm, as the occurrence of smectic layering is more likely here. We recorded diffractograms over the relevant angular range between 385 and 345 K in temperature steps of 0.5 or 1 K, respectively.

An alternative explanation considers the structural specificity of bent‐core molecules, in particular their ability to assemble into splay‐bend structures. The associated positional rearrangement in this case leads to a tighter packing of the molecules and thus increases the macroscopic optical anisotropy. Of course, we assume that the splay‐bend (N_*SB*_) wave is characterized by a short wavelength, and therefore the spatial deviations of the molecular axes from the long channel axis are sufficiently small to ensure that the contribution of the positional reorganization of the molecules to the macroscopic optical birefringence is dominant and the excess retardation is positive. We believe that it is no coincidence that the retardation behavior of the nanoconfined bent mesogen core below *T*_*x*_, especially in the regime of moderate confinement (25 ⩽*D* ⩽ 60 nm), see Figure 4c–f, closely resembles the bulk birefringence behavior exhibited by both the electric field‐induced N_*SB*_ phase and the N_*SB*_ defect wall separating monochiral domains of opposite chirality, as shown by Meyer et al.^[^ ^46^ ^]^ It should be noted that the biaxiality of the confined N_*SB*_ structure, represented at the nanoscale level within the individual channels, is macroscopically averaged at the macroscopic level, so that the nanocomposite membrane remains optically uniaxial.

**Figure 6:**
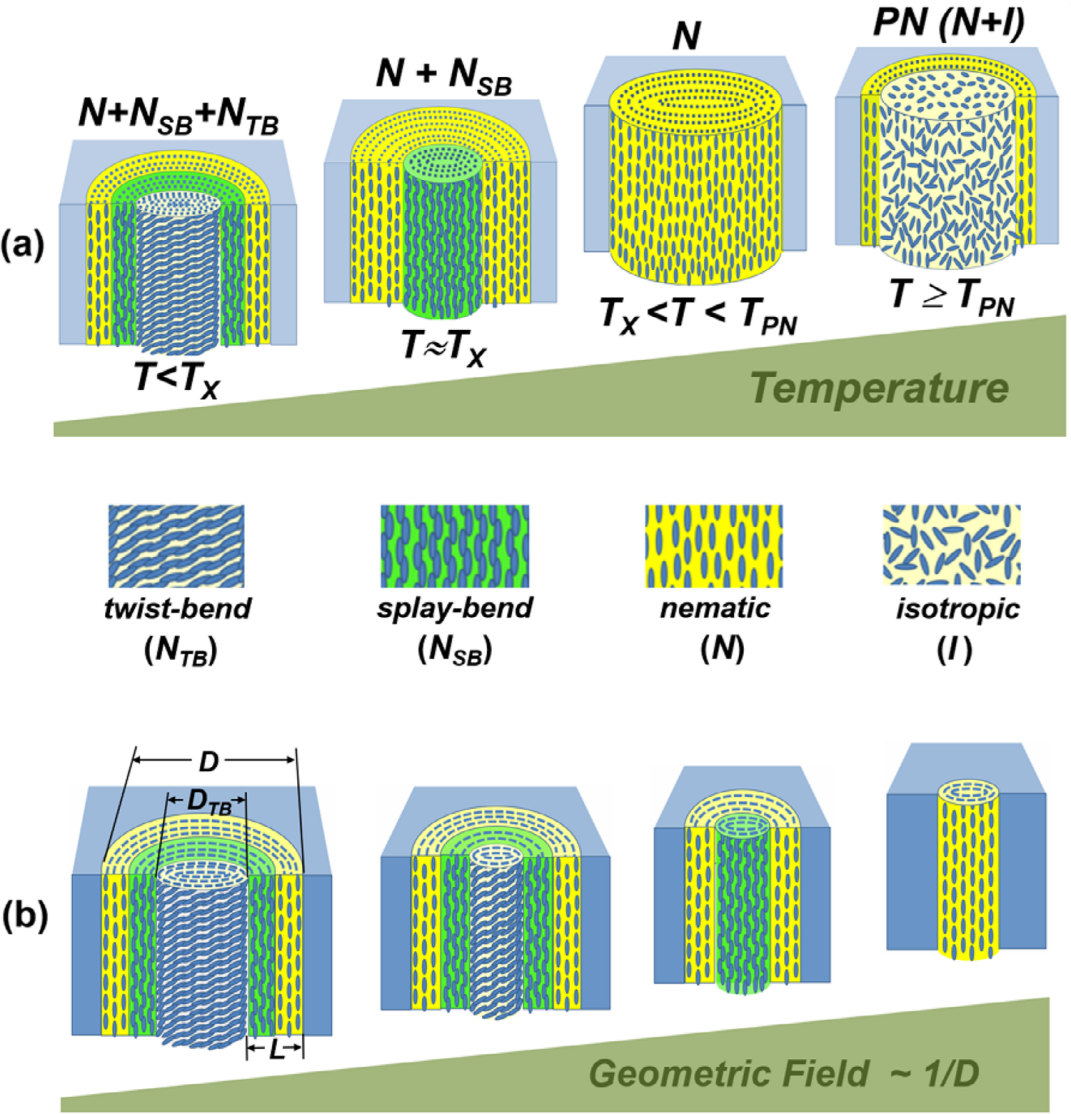
a) Characteristic self‐assembly of the CBC7CB bent‐core nematic as a function of temperature and b) confinement in cylindrical nanopores.

Assuming that the appearance of the N_*SB*_ structure just below *T*_*x*_ is indeed real, its transformation into the N_*TB*_ phase upon cooling is another challenging issue, leading us to basically two different hypothetical scenarios. The first considers the gradual winding of the splay‐bend chains into a heliconic structure upon cooling, which occurs simultaneously throughout the N_*SB*_ core region. An alternative scenario, consistent with the observed optical retardation behavior and its evolution under spatial confinement, assumes the formation of the N_*TB*_ core in the central part of the pore filling with its subsequent radial expansion upon cooling at the expense of the reduction of the intermediate N_*SB*_ shell, as schematically shown in **Figure** **6**a. According to this hypothesis, the bent‐core CB7CB mesogen embedded in the cylindrical channel of sufficiently large diameter constitutes a hybrid three‐phase system consisting of the interfacial paranematic layer, the N_*SB*_ intermediate shell and the N_*TB*_ core. Accordingly, under weak confinement conditions, i.e., for large channel diameters, the N_*TB*_ phase component, due to its relatively large volume fraction, provides the dominant contribution to the optical anisotropy, so that the excess retardation well below *T*_*x*_ is essentially negative, i.e., Δ_*x*_ < 0, see e.g., Figure 4a–d. Surprisingly, the intermediate layer N_*SB*_ can be considered as a defective cylindrical wall between the paranematic layer and the N_*TB*_ core, somewhat similar to the N_*SB*_‐defective wall between two adjacent N_*TB*_ domains of opposite polarity studied in Ref. [46]. The existence of the interlayer N_*SB*_ is thermodynamically unavoidable, so the reduction of the channel diameter causes the reduction of the N_*TB*_ core, first until its complete disappearance, which happens under actual experimental conditions for channel diameters *D* approximately smaller than 15 nm. Correspondingly, under strong confinement (8 ⩽ *D* ⩽ 12 nm) the excess retardation Δ_*x*_ ⩾ 0 in the entire temperature range below *T*_*x*_, see Figure 4g,i. The remaining N_*SB*_ phase fraction apparently vanishes under stronger spatial confinement, i.e., for channel diameters about 7 nm. The corresponding scaling structure‐phase evolution of the confined bent‐core mesogen is sketched in Figure 6b. The increasing geometric field, which is inversely related to the channel diameter (*σ*∝*D* ^−1^), successively suppresses the N_*TB*_ and N_*SB*_ core fractions. The remaining N phase is largely dominated by the strong paranematic contribution, leading to an extended paranematic tail in the optical retardation behavior (Figure 4j) and a significantly temperature‐stretched PN‐to‐N phase transition, in good agreement with the phenomenological prediction of the KKLZ model.^[^ ^18^, ^19^ ^]^

**Figure 7:**
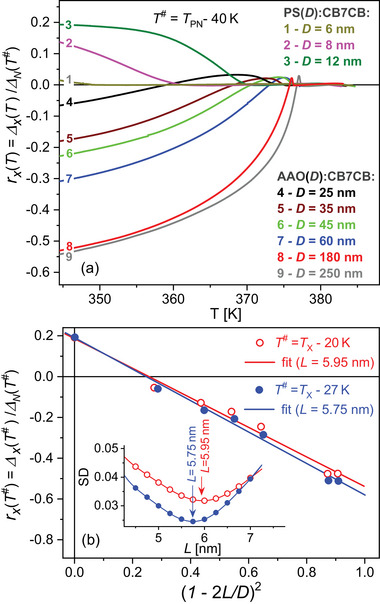
Modeling of optical retardation for the twist bend‐to‐splay bend transition and of the corresponding phase coexistence in nanoconfinement. a) The normalized (dimensionless) excess retardation, $r_{x}\left(T\right)=\Delta_{x}\left(T\right)/\Delta_{N}\left(T^{\#}\right)$ versus *T* in the N_*x*_‐phase. The normalization denominator corresponds the nematic excess retardation $\Delta_{N}\left(T^{\#}\right)$ taken at temperature $T^{\#}=T_{PN}-40$ K, i.e., the value extrapolated to the N_*x*_ multiphase state ($T^{\#}<T_{x}$). b) The normalized excess retardation, $r_{x}\left(T\right)=\Delta_{x}\left(T^{\#}\right)/\Delta_{N}\left(T^{\#}\right)$ vs (1 − 2*L*/*D*)^2^ as extracted from the experimental data (open and solid circles, $T_{x}-T^{\#}=$ 20 and 27 K, see labels) and their linear fits (solid lines). The heterobilayer thickness *L* was set to be a variable fitting parameter and has been determined by minimization of the standard deviation (SD) within the iterative fitting procedure, see insert in panel (b).

A quantitative analysis of the excess retardation Δ_*x*_(*T*) below *T*_*x*_ gives additional insight into the scaling characterization of the spatial confinement evolution of the N‐N_*SB*_‐N_*TB*_ multiphase system. The measured retardation Δ(*T*) depends on the porosity, refractive index and thickness of the mesoporous host matrices used in the preparation of the nanocomposites, which vary significantly, making a direct quantitative comparison of the extracted excess retardation Δ_*x*_(*T*) invalid. However, considering that the excess retardation Δ_*N*_ and Δ_*x*_ scale in the same way with the morphological and geometrical characteristics of the host membranes and associated nanocomposites, the quantitative analysis can be rationalized by considering the normalized dimensionless excess retardation, $r_{x}\left(T\right)=\Delta_{x}\left(T\right)/\Delta_{N}\left(T^{\#}\right)$, where the normalization denominator corresponds to the nematic phase extrapolated excess retardation value $\Delta_{N}\left(T^{\#}\right)$ taken at temperature $T^{\#}=T_{PN}-\Delta T<T_{x}$. **Figure** **7**a shows the *r*_*x*_(*T*) dependence ($T^{\#}=T_{PN}-40$ K) for the series of *AAO*:CB7CB and *PS*:CB7CB nanocomposites.

We can show that the observed structural evolution of the bent‐core mesogen CB7CB under confinement can be interpreted by considering a simple geometric model which assumes the geometric reduction of the N_*TB*_‐core with a decrease of the channel diameter at a constant thickness *L* of the N_*SB*_/*N* heterobilayer, as sketched in Figure 6b (left). In nanoscale inhomogeneous media, the macroscopic (averaged) optical anisotropy, quantified by the birefringence Δ*n*, is defined as the sum of the volume fraction polarizability contributions of its locally anisotropic elements. A good approximation is Δ*n*∝Δ*ε* = *V* ^−1^∑_*i*_ *V*_*i*_Δ*ε*_*i*_, where Δ*ε* is the macroscopic optical dielectric anisotropy expressed by its volume fraction local contributions (*V*_*i*_/*V*)Δ*ε*_*i*_. For the sake of simplicity aimed at reducing the number of fitting parameters, we model the *N*_*x*_ state of the confined bent‐core CB7CB LC by the two‐component system consisting of the cylindrical N_*TB*_ core and the N_*SB*_/N heterobilayer, whose local dielectric optical anisotropy is described by the effective constants Δ*ε*_*t*_ and Δ*ε*_*ns*_, respectively. It is also assumed that the layer thickness ratio $L_{N_{SB}}/L_{N}$ ($L_{N_{SB}}+L_{N}=L$) is nearly independent of the channel diameter and weakly affects the local dielectric anisotropy Δ*ε*_*ns*_, which remains valid as long as *L* < <*D*. The dimensionless excess retardation at a given temperature in the N_*x*_ state ($T=T^{\#}<T_{x}$) can be represented as follows:

(3) $$\begin{array}{ccc} r_{x}\left(T^{\#},D\right) & = & \frac{\Delta \varepsilon_{t}D_{TB}^{2}+\Delta \varepsilon_{ns}\left(D^{2}-D_{TB}^{2}\right)-\Delta \varepsilon_{n}D^{2}}{\Delta \varepsilon_{n}D^{2}}\\ & = & \frac{\Delta \varepsilon_{ns}-\Delta \varepsilon_{n}}{\Delta \varepsilon_{n}}-\left(\frac{\Delta \varepsilon_{ns}-\Delta \varepsilon_{t}}{\Delta \varepsilon_{n}}\right)\frac{{\left(D-2L\right)}^{2}}{D^{2}}, \end{array}$$

where *D*_*TB*_ is the diameter of the *N*_*TB*_ core (see Figure 6b, left), Δ*ε*_*n*_ is the effective dielectric anisotropy constant of the hypothetical N‐phase. In such a geometrical approach it is assumed that Δ*ε*_*n*_, Δ*ε*_*ns*_ and Δ*ε*_*t*_, which depend on temperature, are independent of *D*. Accordingly, $r_{x}\left(T^{\#},D\right)=C_{1}+C_{2}\cdot {\left(1-2L/D\right)}^{2}$, where *C*_1_ = (Δ*ε*_*ns*_ − Δ*ε*_*n*_)/Δ*ε*_*n*_, *C*_2_ = (Δ*ε*_*ns*_ − Δ*ε*_*t*_)/Δ*ε*_*n*_, and *L* can be considered as fitting parameters of the model. The goal of our analysis was to extract the effective thickness *L* of the N_*SB*_/N heterobilayer by linearly fitting the experimentally derived dependences $r_{x}\left(T^{\#},D\right)$ versus (1 − 2*L*/*D*)^2^, see Figure 7b.

The geometric model fits the experimental data reasonably well, as can be seen from the two sets of data points corresponding to the temperatures $T^{\#}=20\;K$ and $T^{\#}=27\;K$ chosen for analysis. In the linear fitting procedure, the heterobilayer thickness *L* was set as a variable parameter, while its fit value was derived by minimizing the standard deviation (SD) with respect to *L* extracted in a series of successive linear fits. The minimum of the nearly parabolic dependence SD(*L*) (Figure 7b, insert) corresponds to the effective heterobilayer thickness value, which differs insignificantly for the analyzed temperatures in the N_*x*_ state and appears in the range of 5.7–5.9 nm. Geometrically it follows that for *D*_*TB*_ → 0 at *D* → 2*L* the N_*TB*_ phase fraction seems to be completely suppressed for channel diameters of about 12 nm, which seems to be in agreement with the experiment. It is also important to note that both linear fits $r_{x}\left(T^{\#},D\right)$ at 1 − 2*L*/*D* = 0 approach the experimentally derived normalized excess retardation value $r_{x}\left(T^{\#},D=12\;\text{nm}\right)$ for the *PS*(12 nm):CB7CB nanocomposite sample, which is essentially positive, indicating the dominant contribution of the N_*SB*_‐fraction.

Taken together, the simple geometric confinement model is able to provide a realistic description of the bent‐core nematogen CB7CB under cylindrical nanoconfinement. Increasing geometrical constraint concentrically shrinks the size of the N_*TB*_‐core, similar to what is observed in a number of ferroic materials under external fields or mechanical stress. A typical example is the displacement of domain walls observed in incommensurate ferroelectrics under the influence of an electric field^[^ ^62^ ^]^ or in incommensurate ferroelastic crystals under the influence of uniaxial compression,^[^ ^63^ ^]^ causing the expansion of domain regions with a specific polarization or deformation at the expense of shrinking the opposite domains. In the case of a confined bent nematic core, the spatial constraint can be associated with a geometric field, so by varying it one can shift the intermediate cylindrical defect N_*SB*_‐layer (i.e., cylindrical wall), thus expanding or reducing the size of the core N_*TB*_‐domain. The comparison of the birefringence behavior of the CB7CB mesogen in the N_*x*_ phase under cylindrical confinement, investigated in the present study, with its bulk birefringence behavior under an applied electric field along the helical axis (*E*||*z*), reported in Ref. [46], is striking. In the regime of weak confinement (*D* = 180, 250 nm), the optical birefringence behaves practically identical to the electrically free bent‐core mesogen embedded in a few micrometer parallel plate glass cell.

Under strong confinement (e.g., *D* = 25 ‐ 45 nm), its behavior is similar to that in the bulk state under the applied strong electric field (*E* = 8 V/µm), which is interpreted as a field‐induced N_*SB*_ phase according to.^[^ ^46^ ^]^ Our study, on the other hand, clearly shows that in the regime of strong confinement the bent‐core mesogen in the N_*x*_ state still represents a multiphase formation of coexisting N, N_*SB*_, and N_*TB*_ phase fractions, and that stronger spatial confinement (*D* ⩽ 12 nm) is required to completely suppress the N_*TB*_ phase. It is expected that the bulk experiments at much higher electric fields could also reproduce such behavior, although their practical realization may be challenging due to the possible electrical breakdown of the bulk LC layer.

## 4 Conclusion

This study examines how the bent‐core liquid crystal dimer CB7CB organizes under confinement within mesoporous alumina and silica substrates featuring cylindrical nanochannels (diameters: nanometers to hundreds of nanometers). Optical polarimetry reveals that weak confinement stabilizes a heterophase system with concentric N (paranematic), N_SB_ (smectic‐like), and N_TB_ (twist‐bend) phases. Stronger confinement progressively suppresses N_TB_ and N_SB_, leaving only the disordered N phase. Intriguingly, geometric confinement mimics electric field effects on optical birefringence, suggesting analogous mechanisms for manipulating the molecular order.

Overall, our study demonstrates how the self‐assembly behavior and thus the optical properties of bent‐core nematics can be systematically tailored by confinement. Moreover, it is a fine example of how embedding liquid crystals in nanoporous solids allows one to synthesize nanocomposites with tunable and temperature‐sensitive optical anisotropy.

In practice, our nanocomposites based on mesoporous alumina (AAO) or silica (PS) are macroscopic membranes, typically 0.1–0.3 mm thick, with lateral dimensions determined by the size of the nanoporous host. For mesoporous silica, membrane size is limited by the silicon wafers used in fabrication (up to ∼300 mm in diameter), while AAO membranes, produced via electrochemical anodization of aluminum, can reach sizes comparable to or larger than a sheet of an A4 paper. Thus, these nanocomposites are suitable for macroscopic applications.

These insights hold considerable implications for the development of next‐generation photonic and electro‐optic materials. By exploiting nanoscale confinement as a tool to tailor liquid crystal phases and their optical responses, it becomes possible to engineer hybrid nanostructures with tunable anisotropy, fast switching behavior, and novel electro‐optic properties. Such structures may find application in advanced optical modulators, sensors, and reconfigurable metamaterials, where precise control over molecular orientation and phase composition is essential. Furthermore, our study provides a framework for understanding how confinement geometry can serve as a design parameter for the rational development of responsive soft matter systems, bridging the gap between fundamental mesophase science and applied nanotechnology.

In the future, studying polar nematic liquid crystals, also known as ferroelectric nematics, could be particularly interesting. These crystals exhibit spontaneous electric polarization, which can be controlled by an external electric field, as discussed in Ref. [64, 65]. For this reason, these materials are unique compared to conventional apolar nematics and have broad prospects for electro‐optical and non‐linear optical applications.^[^ ^64^, ^66^ ^]^ Some ferroelectric liquid crystals can form helical structures,^[^ ^67^ ^]^ such as those exhibited by twist‐bend nematics. Direct polarimetric studies of ferronematic nanocomposites could provide valuable insight into molecular ordering within nanochannels and the effects of confinement at the nanoscale. To our knowledge, there are currently no systematic studies on this topic in the literature, and we consider it a promising area for future research.

## Conflict of Interest

The authors declare no conflict of interest.

## Author Contributions

The manuscript was written through the contributions of all authors. All authors have approved the final version of the manuscript.

## Data Availability

The data that support the findings of this study are available from the corresponding author upon reasonable request.

## References

[1] K. Sentker , A. Yildirim , M. Lippmann , A. W. Zantop , F. Bertram , T. Hofmann , O. H. Seeck , A. Kityk , V, M. G. Mazza , A. Schoenhals , P. Huber , Nanoscale 2019, 11, 23304. 31788679 10.1039/c9nr07143a

[2] G. Chahine , A. V. Kityk , K. Knorr , R. Lefort , M. Guendouz , D. Morineau , P. Huber , Phys. Rev. E 2010, 81, 031703. 10.1103/PhysRevE.81.031703 20365747

[3] G. Chahine , A. V. Kityk , N. Demarest , F.Jean, K. Knorr , P. Huber , R. Lefort , J.‐M. Zanotti , D. Morineau , Phys. Rev. E 2010, 82, 011706. 10.1103/PhysRevE.82.011706 20866634

[4] C. V. Cerclier , M. Ndao , R. Busselez , R. Lefort , E. Grelet , P. Huber , A. V. Kityk , L. Noirez , A. Schoenhals , D. Morineau , J. Phys. Chem. C 2012, 116, 18990.

[5] P. Huber , M. Busch , S. Calus , A. V. Kityk , Phys. Rev. E 2013, 87, 042502. 10.1103/PhysRevE.87.042502 23679431

[6] S. Calus , A. V. Kityk , P. Huber , Microporous Mesoporous Mater. 2014, 197, 26.

[7] S. Calus , A. V. Kityk , L. Borowik , R. Lefort , D. Morineau , C. Krause , A. Schoenhals , M. Busch , P. Huber , Phys. Rev. E 2015, 92, 012503. 10.1103/PhysRevE.92.012503 26274191

[8] C. Grigoriadis , H. Duran , M. Steinhart , M. Kappl , H. J. Butt , G. Floudas , ACS Nano 2011, 5, 9208. 21974835 10.1021/nn203448c

[9] S. H. Ryu , D. K. Yoon , ACS Appl. Mater. Interfaces 2017, 9, 25057. 28677393 10.1021/acsami.7b07693

[10] M. Spengler , R. Y. Dong , C. A. Michal , W. Y. Hamad , M. J. MacLachlan , M. Giese , Adv. Funct. Mater. 2018, 28, 1800207.

[11] P. Huber , J. Phys.: Condens. Matter 2015, 27, 103102. 25679044 10.1088/0953-8984/27/10/103102

[12] P. Huber , K. Sentker , M. Busch , A. V. Kityk , in Soft Matter And Biomaterials On The Nanoscale: The Wspc Reference On Functional Nanomaterials‐Part I (In 4 Volumes), chapter 11, (ed.: P. Huber ) World Scientific Publishing, Singapore, 2020, pp. 377–434.

[13] A. V. Kityk , M. Nowak , M. Reben , P. Pawlik , M. Lelonek , A. Andrushchak , Y. Shchur , N. Andrushchak , P. Huber , Nanoscale 2021, 13, 18714. 34739018 10.1039/d1nr04282c PMC8601124

[14] K. Waszkowska , P. Josse , C. Cabanetos , P. Blanchard , B. Sahraoui , D. Guichaoua , I. Syvorotka , O. Kityk , R. Wielgosz , P. Huber , A. V. Kityk , Optics Letters 2021, 46, 845. 33577526 10.1364/OL.416948

[15] P. Sheng , Phys. Rev. Lett. 1976, 37, 1059.

[16] A. Poniewierski , T. J. Sluckin , Liq. Cryst. 1987, 2, 281.

[17] H. Yokoyama , J. Chem. Soc., Faraday Trans. II 1988, 84, 1023.

[18] Z. Kutnjak , S. Kralj , G. Lahajnar , S. Zumer , Phys. Rev. E 2003, 68, 021705. 10.1103/PhysRevE.68.021705 14524991

[19] Z. Kutnjak , S. Kralj , G. Lahajnar , S. Zumer , Phys. Rev. E 2004, 70, 051703. 10.1103/PhysRevE.70.051703 15600636

[20] A. V. Kityk , M. Wolff , K. Knorr , D. Morineau , R. Lefort , P. Huber , Phys. Rev. Lett. 2008, 101, 187801. 18999865 10.1103/PhysRevLett.101.187801

[21] A. V. Kityk , P. Huber , Phys. Rev. Lett. 2010, 97, 153124.

[22] S. Calus , D. Rau , P. Huber , A. V. Kityk , Phys. Rev. E 2012, 86, 021701. 10.1103/PhysRevE.86.021701 23005774

[23] S. Calus , B. Jablonska , M. Busch , D. Rau , P. Huber , A. V. Kityk , Phys. Rev. E 2014, 89, 062501. 10.1103/PhysRevE.89.062501 25019799

[24] A. V. Kityk , M. Busch , D. Rau , S. Calus , C. V. Cerclier , R. Lefort , D. Morineau , E. Grelet , C. Krause , A. Schoenhals , B. Frick , P. Huber , Soft Matter 2014, 10, 4522. 24832498 10.1039/c4sm00211c

[25] R. Zhang , X. Zeng , M. Prehm , F. Liu , S. Grimm , M. Geuss , M. Steinhart , C. Tschierske , G. Ungar , ACS Nano 2014, 8, 4500. 24758721 10.1021/nn406368e

[26] K. Sentker , A. W. Zantop , M. Lippmann , T. Hofmann , O. H. Seeck , A. V. Kityk , A. Yildirim , A. Schoenhals , M. G. Mazza , P. Huber , Phys. Rev. Lett. 2018, 120, 067801. 29481274 10.1103/PhysRevLett.120.067801

[27] A. Yildirim , K. Sentker , G. J. Smales , B. R. Pauw , P. Huber , A. Schönhals , Nanoscale Adv. 2019, 1, 1104. 36133215 10.1039/c8na00308d PMC9473266

[28] S. Calus , M. Busch , A. V. Kityk , W. Piecek , P. Huber , J. Phys. Chem. C 2016, 120, 11727.

[29] M. Bengoechea , F. Aliev , J. Non‐Cryst. Solids 2005, 351, 2685.

[30] F. M. Aliev , E. F. Arroyo , V. Dolidze , J. Non‐Cryst. Solids 2010, 356, 657.

[31] S. Calus , A. V. Kityk , M. Eich , P. Huber , Soft Matter 2015, 11, 3176. 25759093 10.1039/c5sm00108k

[32] S. Calus , L. Borowik , A. V. Kityk , M. Eich , M. Busch , P. Huber , J. Non‐Cryst. Solids 2015, 17, 22115. 10.1039/c5cp03039k 26255586

[33] M. Busch , A. V. Kityk , W. Piecek , T. Hofmann , D. Wallacher , S. Calus , P. Kula , M. Steinhart , M. Eich , P. Huber , Nanoscale 2017, 9, 19086. 29199756 10.1039/c7nr07273b

[34] R. B. Meyer , in *Molecular Fluids*, volume vol. XXV‐1973 of Les Houches Summer School in Theoretical Physics, (Eds.: R. Balian , G. Weill ), Gordon and Breach, New York, 1976, pp. 273–373.

[35] I. Dozov , Europhys. Lett. 2001, 56, 247.

[36] T. Niori , T. Sekine , J. Watanabe , T. Furukawa , H. Takezoe , J. Mater. Chem. 1996, 6, 1231.

[37] R. A. Reddy , C. Tschierske , J. Mater. Chem. 2006, 16, 907.

[38] H. Takezoe , Y. Takanishi , Jpn. J. Appl. Phys. 2006, 45, 597.

[39] M. Cestari , S. Diez‐Berart , D. A. Dunmur , A. Ferrarini , M. R. de la Fuente , D. J. B. Jackson , D. O. Lopez , G. R. Luckhurst , M. A. Perez‐Jubindo , R. M. Richardson , J. Salud , B. A. Timimi , H. Zimmermann , Phys. Rev. E 2011, 84, 031704. 10.1103/PhysRevE.84.031704 22060387

[40] D. Chen , J. H. Porada , J. B. Hooper , A. Klittnick , Y. Shen , M. R. Tuchband , E. Korblova , D. Bedrov , D. M. Walba , M. A. Glaser , J. E. Maclennan , N. A. Clark , Proc. Natl. Acad. Sci. USA 2013, 110, 15931. 24006362 10.1073/pnas.1314654110 PMC3791744

[41] D. A. Paterson , J. P. Abberley , W. T. Harrison , J. M. Storey , C. T. Imrie , Liq. Cryst. 2017, 44, 127.

[42] P. A. Henderson , C. T. Imrie , Liq. Cryst. 2011, 38, 1407.

[43] E. Forsyth , D. A. Paterson , E. Cruickshank , G. J. Strachan , E. Gorecka , R. Walker , J. M. Storey , C. T. Imrie , J. Mol. Liq. 2020, 320, 114391.

[44] R. Memmer , Liq. Cryst. 2002, 29, 483.

[45] S. M. Shamid , S. Dhakal , J. V. Selinger , Phys. Rev. E 2013, 87, 052503. 10.1103/PhysRevE.87.052503 23767556

[46] C. Meyer , C. Blanc , G. R. Luckhurst , P. Davidson , I. Dozov , Sci. Adv. 2020, 6, eabb8212. 32917595 10.1126/sciadv.abb8212 PMC7467706

[47] G. Pająk , L. Longa , A. Chrzanowska , Proc. Natl. Acad. Sci. USA 2018, 115, E10303. 30309960 10.1073/pnas.1721786115 PMC6217413

[48] K. Merkel , A. Kocot , J. K. Vij , G. Shanker , Phys. Rev. E 2018, 98, 022704. 30253534 10.1103/PhysRevE.98.022704

[49] A. Varanytsia , L.‐C. Chien , Sci. Rep. 2017, 7, 41333. 28117429 10.1038/srep41333 PMC5259770

[50] I. Dozov , C. Meyer , Liq. Cryst. 2017, 44, 4.

[51] P. Karbowniczek , M. Cieśla , L. Longa , A. Chrzanowska , Liq. Cryst. 2017, 44, 254.

[52] R. You , W. Park , E. Carlson , S. H. Ryu , M. J. Shin , E. Guzman , H. Ahn , T. J. Shin , D. M. Walba , N. A. Clark , D. K. Yoon , Liq. Cryst. 2019, 46, 316.

[53] B. Robles‐Hernández , N. Sebastián , M. R. de la Fuente , D. O. López , J. Salud , S. Diez‐Berart , Liq. Cryst. 2024, 51, 1537.

[54] Quantum chemical calculation using semiempirical AM1 method (HyperChem‐8.0) gives for the equilibrium bent‐shaped conformation of CB7CB the following polarizability constans: *α* _ *xx* _ = 175 a.u., *α* _ *yy* _ = 320 a.u. and *α* _ *zz* _ = 465 a.u.

[55] G. Babakhanova , Z. Parsouzi , S. Paladugu , H. Wang , Y. A. Nastishin , S. V. Shiyanovskii , S. Sprunt , O. D. Lavrentovich , Phys. Rev. E 2017, 96, 062704. 29347367 10.1103/PhysRevE.96.062704

[56] M. P. Kumar , J. Karcz , P. Kula , S. Dhara , Phys. Rev. Mater. 2021, 5, 115605.

[57] D. O. López , N. Sebastian , M. R. de la Fuente , J. C. Martínez‐García , J. Salud , M. A. Pérez‐Jubindo , S. Diez‐Berart , D. A. Dunmur , G. R. Luckhurst , J. Chem. Phys. 2012, 137, 034502. 22830706 10.1063/1.4733561

[58] V. Borshch , Y. K. Kim , J. Xiang , M. Gao , A. Jákli , V. P. Panov , J. K. Vij , C. T. Imrie , M. G. Tamba , G. H. Mehl , O. D. Lavrentovich , Nat. Commun. 2013, 4, 2635. 24189583 10.1038/ncomms3635 PMC3831290

[59] S. Gruener , P. Huber , J. Phys.: Condens. Matter 2011, 23, 184109. 21508488 10.1088/0953-8984/23/18/184109

[60] M. R. Tuchband , M. Shuai , K. A. Graber , D. Chen , C. Zhu , L. Radzihovsky , A. Klittnick , L. Foley , A. Scarbrough , J. H. Porada , M. Moran , J. Yelk , J. B. Hooper , X. Wei , D. Bedrov , C. Wang , E. Korblova , D. M. Walba , A. Hexemer , J. E. Maclennan , M. A. Glaser , N. A. Clark , Crystals 2024, 14, 583.

[61] Z. Li , A. Raab , M. A. Kolmangadi , M. Busch , M. Grunwald , F. Demel , F. Bertram , A. V. Kityk , A. Schönhals , S. Laschat , P. Huber , ACS Nano 2024, 18, 14414. 38760015 10.1021/acsnano.4c01062 PMC11155240

[62] J. Holakovsky , V. Dvodrak , J. Phys. C: Solid State Phys. 1988, 21, 5449.

[63] A. Kityk , V. Soprunyuk , A. Fuith , W. Schranz , H. Warhanek , Phys. Rev. B 1996, 53, 6337. 10.1103/physrevb.53.6337 9982031

[64] H. Nishikawa , K. Shiroshita , H. Higuchi , Y. Okumura , Y. Haseba , S.‐i. Yamamoto , K. Sago , H. Kikuchi , Adv. Mater. 2017, 29, 1702354. 10.1002/adma.201702354 29023971

[65] X. Chen , E. Korblova , D. Dong , X. Wei , R. Shao , L. Radzihovsky , M. A. Glaser , J. E. Maclennan , D. Bedrov , D. M. Walba , N. A. Clark , Proc. Natl. Acad. Sci. USA 2020, 117, 14021. 32522878 10.1073/pnas.2002290117 PMC7322023

[66] J. Li , H. Nishikawa , J. Kougo , J. Zhou , S. Dai , W. Tang , X. Zhao , Y. Hisai , M. Huang , S. Aya , Sci. Adv. 2021, 7, eabf5047. 33883139 10.1126/sciadv.abf5047 PMC8059932

[67] J. Karcz , J. Herman , N. Rychłowicz , P. Kula , E. Górecka , J. Szydlowska , P. W. Majewski , D. Pociecha , Science 2024, 384, 1096. 38843325 10.1126/science.adn6812

